# Supplementary material for: Between Innate and Adaptive Immune Responses: NKG2A, NKG2C, and CD8+ T Cell Recognition of HLA-E Restricted Self-Peptides Acquired in the Absence of HLA-Ia
Source: Int J Mol Sci. 2019 Mar 22;20(6):1454. doi: 10.3390/ijms20061454 (PMC6471057; doi:10.3390/ijms20061454)
Supplement: Supplementary file 1 [file ijms-20-01454-s001.pdf]

Supplementary Figures

Between innate and adaptive immune responses: HLA-E restricted self-peptides acquired during an artificial hCMV infection determine the cell fate

Wiebke C. Pump[1]*, Thomas Kraemer[1]*, Trevor Huyton[2], Gia-Gia T. Hò[1], Rainer Blasczyk[1], Christina Bade-Döding[1]

* these authors contributed equally to this work

[1]Institute for Transfusion Medicine, Hannover Medical School, Hannover, Germany

[2]Department of Cellular Logistics, Max Planck Institute for Biophysical Chemistry, Am Fassberg 11, 37077 Göttingen, Germany

Supplementary Figures

Supplementary Fig. 1 Native PAGE of purified sNKG2/CD94 heterodimers

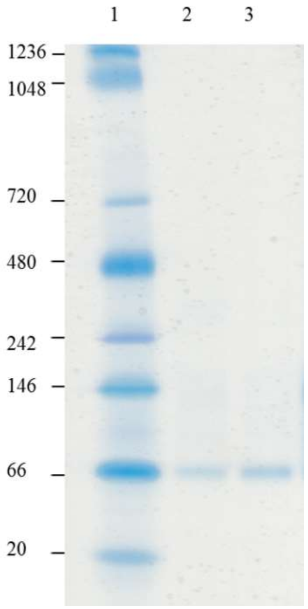

Lanes: 1, standard proteins sized in kDa at left; 2, sNKG2A/CD94; 3, sNKG2C/CD94. Purified sNKG2/CD94 heterodimers exhibit a molecular mass about ~ 66 kDa and no detectable monomers after purification. Proteins were detected with Coomassie blue G-250.

Supplementary Fig. 2 HLA-E surface expression on T2E cells

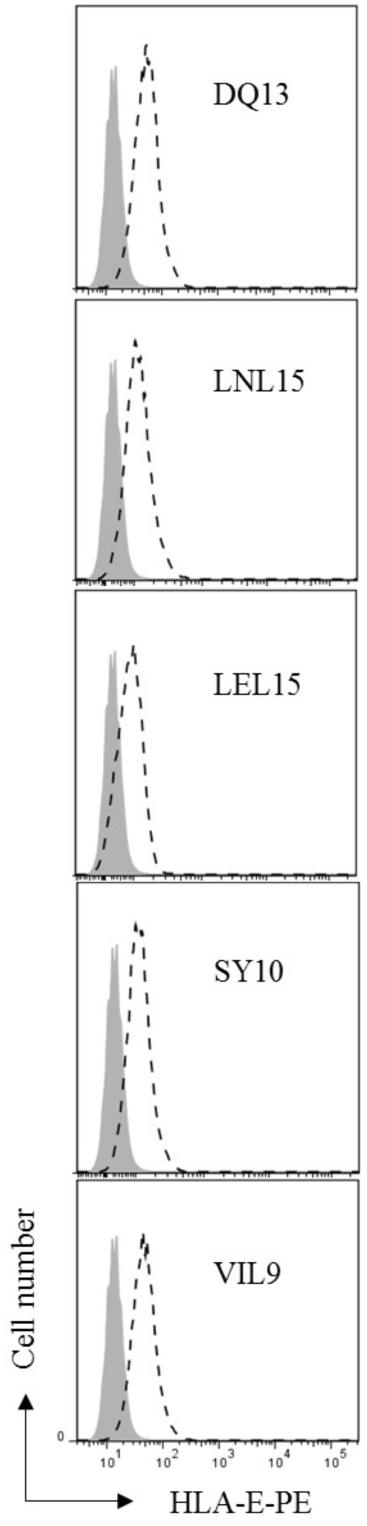

Histograms of HLA-E surface expression levels on T2E cells after incubation with the test peptides. HLA-E surface expression was detected after incubation with the anti-HLA-E 3D12-PE mab and differences in surface expression are indicated: filled grey: T2E cells without peptide, dashed graph: T2E cells with peptide

Supplementary Fig. 3 NKG2/CD94 receptor binding to the p:HLA-E complexes

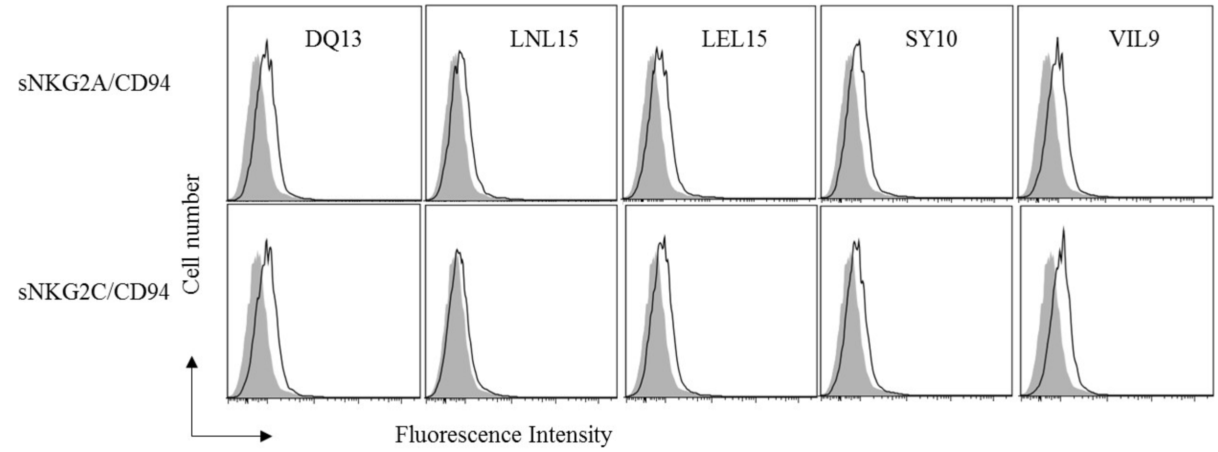

Receptor binding to T2E cells that were not pre-pulsed with peptide served as a negative control and are depicted by the filled grey graph and binding to T2E cells with peptide is indicated by the black graph.

Supplementary Fig. 4 Gating strategy for determination of CD8$^+$ T cell proliferation and phenotype.

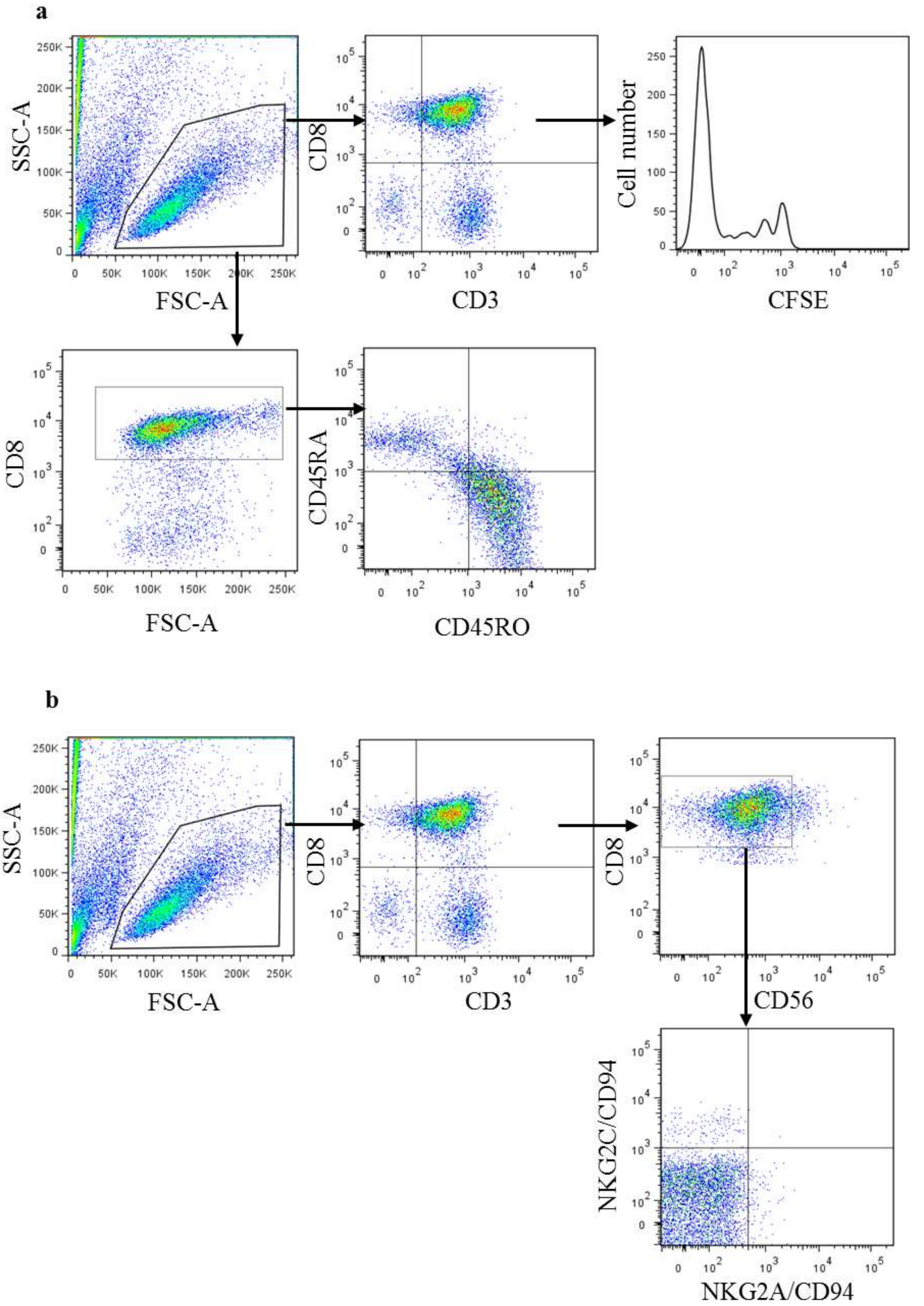

a) Proliferated CD8$^+$ T cells were first gated on live cells (FSC-A versus SSC-A), followed by identification of CD3$^+$CD8$^+$ T cells (CD3 versus CD8). Prolifertaion of CD8$^+$ T cells was analyzed by CFSE dilution. Subgating from live cells to identify CD8$^+$ T cells (FSC-A versus CD8) leads to the determination of CD45RA and CD45RO phenotype of CD8$^+$ T cells (CD45RO versus CD45RA). b) Gating from live cells to identify CD3$^+$CD8$^+$ T cells. CD3$^+$CD8$^+$ T cells were strictly gated on CD56$^-$ cells and NKG2A/CD94 and NKG2C/CD94 phenotype of CD8$^+$ T cells were determined (NKG2A/CD94 versus NKG2C/CD94).

Supplementary Fig. 5 Live/Dead discrimination of proliferated CD8^{+} T cells.

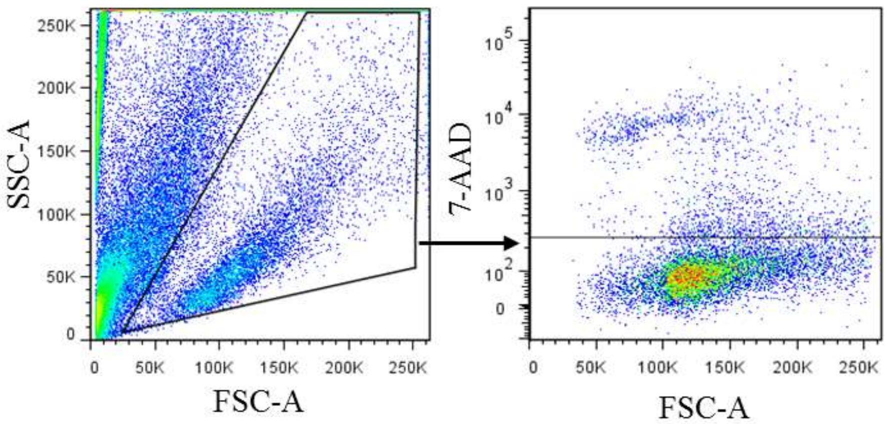

Gating on live cells (FSC-A versus SSC-A) was verified by 7-AAD staining (FSC-A versus 7-AAD) that represents the subgated population to determine the CD3^{+}CD8^{+} population.
